# INDACO project: COPD and link between comorbidities, lung function and inhalation therapy

**DOI:** 10.1186/2049-6958-10-4

**Published:** 2015-01-27

**Authors:** Giorgio Fumagalli, Fabrizio Fabiani, Silvia Forte, Massimiliano Napolitano, Giovanni Balzano, Matteo Bonini, Giuseppe De Simone, Salvatore Fuschillo, Antonella Pentassuglia, Franco Pasqua, Pietro Alimonti, Stefano Carlone, Claudio M Sanguinetti

**Affiliations:** Pulmonary Departments, San Filippo Neri General Hospital, Rome, Italy; San Giovanni-Addolorata General Hospital, Rome, Italy; San Giovanni Battista Hospital, Rome, Italy; Quisisana Clinical Center, Rome, Italy; Pneumology Rehabilitation, Villa delle Querce Hospital, Nemi, Rome, Italy; Pulmonary Rehabilitation Unit, Salvatore Maugeri Foundation, Telese Terme, Italy; Department of Public Health and Infectious Diseases, “La Sapienza” University of Rome, Rome, Italy; Department of Pneumology, Villa Margherita, Rehabilitation Institute of Benevento, Benevento, Italy; Pulmonary Department, San Pietro Hospital, Rome, Italy; UOC Pneumologia, A.C.O. San Filippo Neri, Via Martinotti, 20, 00135 Rome, Italy

**Keywords:** BMI, Charlson Index, Comorbidities, COPD, COPD exacerbation, FEV1, Inhaled therapy, Smoking

## Abstract

**Background:**

Chronic Obstructive Pulmonary Disease (COPD) is characterized by respiratory and extrarespiratory components referring both to systemic complications of COPD, like skeletal muscle myopathy, weight loss and others, and frequently associated comorbidities, interesting various organs and systems (cardiovascular diseases, malignancies, osteoporosis, diabetes, etc.). These comorbidities may increase the rate of hospitalization of COPD patients and have a huge effect on the outcomes of the respiratory disease. Inhalation therapy of COPD with bronchodilators and steroid is primary driven by airflow obstruction, symptoms like dyspnoea, and acute exacerbations. INDACO project has been developed in 2013 to assess the prevalence and type of comorbidities in COPD patients referred to the outpatient wards of some hospitals in Central and South Italy and a preliminary report has recently been published. In the present study, after widening that database, we evaluate the prevalence of comorbidities and the relationships between comorbidities and sex, age, symptoms, lung function and inhalation therapy in COPD patients.

**Methods:**

In each enrolled patient, anthropometric and anamnestic data, smoking habits, respiratory function, GOLD (Global initiative for Chronic Obstructive Lung Disease) severity stage, Body Mass Index (BMI), number of acute COPD exacerbations in previous years, presence and type of comorbidities, and the Charlson Comorbidity Index (CCI) were recorded.

**Results:**

We collected data of 569 patients (395 males and 174 females, mean age 73 ± 8.5 yrs). The prevalence of patients with comorbidities was 81.2%. Overall number of comorbidities was not related to airflow obstruction and age, but to acute exacerbation of COPD, dyspnoea measured with MRC scale, and male gender. A subgroup analysis revealed that ischaemic heart disease was predominant in males, whereas mood disorders in females. The use of a more complex (multi-drug) inhalation therapy was related with bronchial obstruction measured by FEV_1_/FVC (p for trend = 0.003) and number of comorbidities (p for trend = 0.001). In multivariate analysis, only airflow obstruction and number of comorbidities were determinant of complexity of therapy, but not MRC and acute exacerbation of COPD. However, the statistical model reached an extreme low degree of significance (r^2 = 0.07).

**Conclusions:**

Our study showed a high prevalence of comorbidities in COPD, with some differences related to gender. Number of comorbidities and airflow obstruction represent the determinant of inhalation therapy prescription. Dyspnoea and acute exacerbation of COPD, unlikely suggested by guidelines, are not significant drivers of therapy in the real life setting of our study.

## Background

Chronic obstructive pulmonary disease (COPD) is an important cause of morbidity and mortality around the world and represents a social and economic challenge for national health systems [1, 2]. Airflow limitation, that is not fully reversible due to airways inflammation and remodelling, and to parenchymal destruction, is the greater cause of disability and mortality in COPD patients. Moreover, the presence of comorbidities is related to higher mortality, overconsumption of drugs and worst quality of life [3].

Several attempts have been made to define an index of COPD and comorbidities severity, such as BODE [4] and COTE [5]. However, the relationship between COPD and comorbidities and inhalation therapy has not been evaluated in real life settings.

In 2013, we published the INDACO project to evaluate the prevalence of comorbidities in COPD patients referred to pulmonary units of four major general hospitals in Rome, and to search correlations between prevalence and type of comorbidities and patients’ clinical and respiratory function characteristics [6]. That study analysed 169 patients. We found a high prevalence of comorbidities in our population, a significant correlation between some comorbidities and COPD and its acute exacerbations, and a significant correlation between inhalation therapy, functional impairment, and a lower prevalence of comorbidities.

This study represents the evolution of the first pilot study and has the aim to confirm the possible relationship among comorbidities, COPD and respiratory inhalation therapy. We decided to enlarge the observation to central area of Italy, to avoid bias related to differences in geographic latitude and in local health system in Regions far from each others.

## Methods

### Study design

This is an observational study, carried out as progression of the INDACO Pilot study, over 12 months of observation, without a control group, with the engagement of further Respiratory Units in a more extended area of Central and South Italy.

Patients of both sexes, aged more than 40 years and with a diagnosis of COPD, set by clinical history, respiratory function tests and radiologic examination, were enrolled in this study. They were part of the outpatient sample, referred either for a first examination or for the follow up to General and University Hospitals and Respiratory Rehabilitation centers. Patients without respiratory function test were excluded from the study, both because they were not able to perform the tests or they had performed them more than one month before. We excluded from our study also patients with other lung diseases but without COPD, because of a substantial difference in respiratory inhalation therapy.

### Data collection

The same specifically-designed computerized questionnaire, already used and described in the pilot study [6], was filled in by a respiratory physician for each COPD patient. Personal and anthropometric data, smoking habit, clinical history of COPD, number of acute exacerbations in the previous year, results of spirometry, arterial blood gas analysis, and 6-minute walking test (6MWT) (performed during clinical examination or within 3 months), degree of dyspnea evaluated with the modified Medical Research Council Dyspnea scale (mMRC), COPD inhalation therapy and domestic drug regimen, were the collected variables. For each patient, comorbidities were identified either on the basis of anamnesis or by drugs use. The Charlson Comorbidity Index (CCI) [7] was calculated in a standard way for each patient. The reported inhalation therapy was double checked for correctness by the lung physician before reporting it in the questionnaire. For the analysis of data, we divided this variable in five classes: 1) “naïve” (no inhaler therapy), 2) one bronchodilator either a long-acting beta-adrenergic (LABA) or a long-acting anti-muscarinic (LAMA), 3) two bronchodilators (LABA plus LAMA) 4) LABA or LAMA and Inhaled Corticosteroids (ICS), 5) LABA plus LAMA plus ICS.

### Statistical analysis

We firstly analysed the distribution of parameters of respiratory function, anthropometric characteristics, acute exacerbations, and comorbidities in relation to gender and inhalation therapy.

Then, we looked for possible correlations between the number of acute exacerbations in the previous year and the level of bronchial obstruction and comorbidities, CCI score, and inhalation therapy. The five above mentioned classes of therapy were graduated on the basis of progressively greater complexity. We evaluated distribution of complexity of inhalation therapy by respiratory function, anthropometric characteristics and comorbidities. Data are reported as mean value ± SD (standard deviation from the mean). The statistical significance was investigated with the analysis of variance for normally distributed groups and p < 0.05 was considered statistically significant. Linear regression was used to obtain a significant model for use of inhalation drugs pursuant to respiratory function variables, respiratory symptoms, comorbidities, and acute exacerbations of COPD (AECOPD).

The Ethics Committee of involved hospitals was informed about this observational study, which required a patients’ informed consent, as usual form of routinely clinical activities.

## Results

From 1^st^ September 2009 to 30^th^ August 2013 we enrolled 569 patients, 395 males and 174 females, mean age 73 ± 8.5 years, all affected by COPD. Anthropometric and respiratory function characteristics are summarized in Table 1, divided by sex (male and female).

**Table 1:** **Sample distribution by sex**

	Male	Female	***p***
Age (mean ± SD)	73(±8)	73(±9)	ns
MRC (mean ± SD)	2.29(±0.9)	2.29(±0.9)	ns
BMI (mean ± SD)	27.2 (±5.1)	26.9(±5.3)	ns
FEV1% pred. (mean ± SD)	52.9(±19.6)	53.5(±17.7)	ns
FEV1/FVC % (mean ± SD)	59.3(±14.9)	60.1(±16.9)	ns
COPD exacerbations (mean ± SD)	1.66(±1.09)	1.48(±0.96)	0.057
Charlson index (mean ± SD)	5.1(±2.2)	4.9(±2.2)	ns
Comorbidities (mean ± SD)	1.14(±1.2)	0.9(±1.2)	0.03

**Figure 1:**
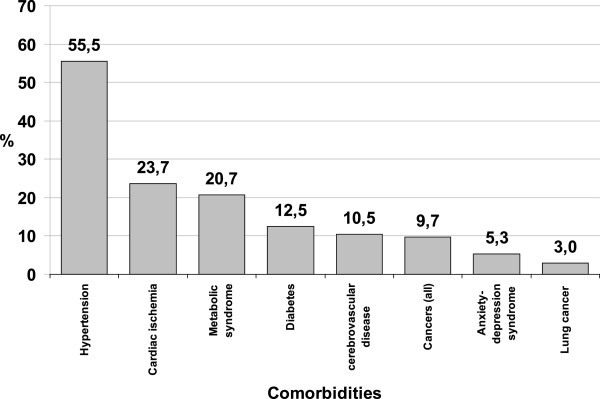
**Prevalence (%) of different comorbidities in a study population of 569 COPD patients.** Global prevalence of observed comorbidities.

Male and female were not different regarding BMI, FEV_1_, and MRC. Episodes of exacerbations of COPD tended to be higher in males than in females, without reaching the statistical significance (p = 0.057). The prevalence of each comorbidity is shown in Figure 1. Patients presenting comorbidities were 462 (81.2%); out of them 221 patients (38.8%) had only one comorbidity; 141 (24.8%) had two comorbidities, and 100 via (17.5%) had 3 or more comorbidities. The prevalence of each comorbidity is shown in Figure 1.

**Figure 2:**
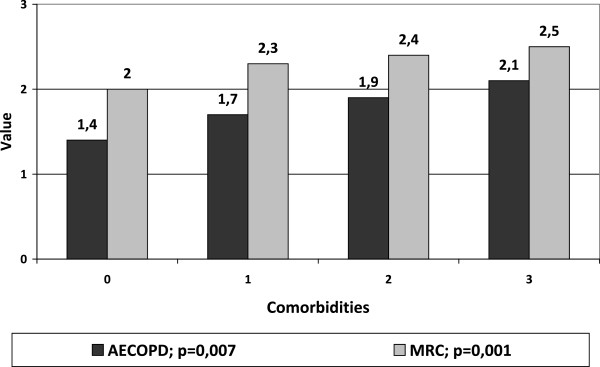
**Values of MRC and COPD exacerbations in relation to the number of comorbidities.** Higher values of MRC and acute exacerbations of COPD in patients with higher prevalence of comorbidities.

Number of comorbidities was not related to airflow obstruction (FEV_1_/FVC and FEV_1_% pred) or age; on the contrary, statistical differences were observed in frequency of acute exacerbation of COPD and mean value of MRC in relation to the number of comorbidities (Figure 2).

The number of comorbidities was different between males and females: 1.14 ± 1.2 and 0.9 ± 1.2, p = 0.03, respectively. Charlson index was similar in both groups: 5.1 ± 2.2 and 4.9 ± 2.1. Moreover, we observed a significant negative correlation between airflow obstruction and prevalence of arterial hypertension and diabetes. Conversely, mood disorders were significantly related to worst respiratory function, while lung cancer was close to statistical significance. The level of bronchial obstruction was not different whether ischaemic heart disease was present or not (Table 2).

**Table 2:** **Mean value of FEV**_**1**_ **/FVC by each comorbidty**

	Presence of comorbidity	Absence of comorbidiy	p
Arterial hypertension	60.8 (±16.1)	57.9 (±14.7)	0.03
Diabetes	64.0 (±15.0)	58.9 (±15.5)	0.01
Ischaemic heart disease	61.2 (±15.5)	59.1 (±15.5)	ns
Mood disorders	51.3 (±17.9)	60.1 (±15.3)	0.003
Lung cancer	59.3 (±7.7)	59.6 (±15.7)	0.054

Among comorbidities, mood abnormality cases were significantly more incident among females than males (8.6% vs 3.8%, RR 0.59, CI 95% 0.40-0.86, p = 0.01). Conversely, cardiovascular ischaemic disease was predominant in males with statistical significance (26.1% vs 18.4%, RR 1.4, CI 95% 1.0-1.9, p = 0.05). No differences among males and females were observed in distribution of hypertension, diabetes, and cancer.

**Figure 3:**
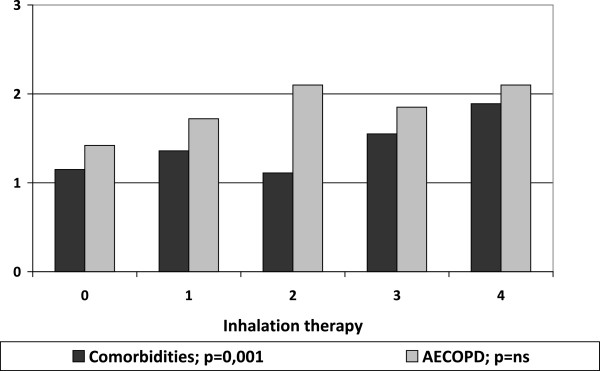
**Mean of comorbidities and COPD acute exacerbations by complexity of inhalation therapy.** Link between comorbidities, acute exacerbations of COPD and greater complexity of inhalation therapy.

**Figure 4:**
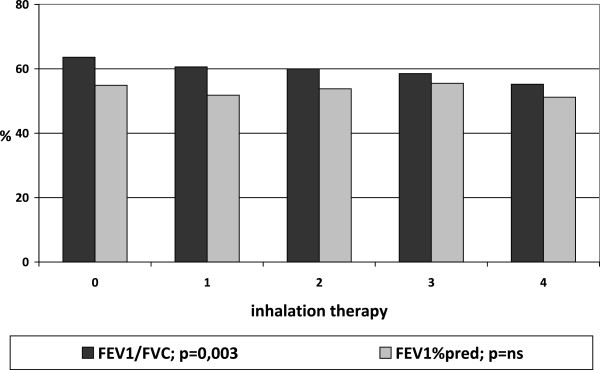
**Airflow obstruction and complexity of inhalation therapy.** Link between comorbidities and respiratory dysfunction evaluated by Tiffeneau Index (FEV_1_/FVC) and FEV_1_ as percent of predicted.

Inhalation therapy was not performed in 107 COPD patients (18.8%), despite the presence of a respiratory obstructive damage (mean value of FEV_1_/FVC = 63.7% ± 14.4; mean value of FEV_1_ % of predicted = 54.9% ± 20.3; mean value of MRC = 2.0 ± 1.1; AECOPD = 1.4 events/year ± 1.3). The inhalation therapy (graduated as already specified in increasing order as: naive, one bronchodilator, two bronchodilators, one bronchodilator plus corticosteroid, two bronchodilators plus corticosteroid) with greater complexity was correlated with the bronchial obstruction measured by FEV_1_/FVC and the number of comorbidities, but not with FEV_1_%pred, acute exacerbations of COPD, age, and BMI (Figures 3 and 4).

With linear regression, we observed a reasonable correlation between complexity of inhalation therapy and MRC, exacerbations of COPD, airflow obstruction and comorbidities (r^2 = 0.07). In our model, only airflow obstruction measured by FEV_1_/FVC and number of comorbidities reached statistical significance (Table 3).

**Table 3:** **Linear regression model with predictors and complexity of inhalation therapy as outcome**

	Coefficient	S.E.	***p***
Comorbidities	0.234	0.057	0.000054
FEV_1_/FVC	-0.013	0.004	0.0019
AECOPD	0.044	0.048	ns
MRC	-0.008	0.073	ns

## Discussion

The frequency of comorbidities in COPD is strictly related to global population health status. Thus, there are many differences in the prevalence of comorbidities in COPD patients, according to geographic area. Based on this observation and differences between international and national studies [8, 9], we designed the INDACO-project with the aim to evaluate the relationships between comorbidities in Italian COPD patients and respiratory dysfunction, smoking habits, BMI, and inhalation therapy. In a pilot project, we previously enrolled and analysed 169 patients in four general hospitals of Rome [6]. On the basis of that study, we extended and improved our research outside the region and besides hospital settings, involving rehabilitation and lower intensity care areas.

In the present study, that includes also the cases previously involved with the same inclusion criteria and analysis methods, we confirmed a higher prevalence of comorbidities in COPD patients (81.2%) compared to national [10] and international data [11]. Updated results demonstrated that there is no correlation between number of comorbidities and the obstructive respiratory dysfunction. Therefore, the higher value of prevalence might be related to a population bias rather than to degree of airflow obstruction.

Among comorbidities, arterial hypertension showed the highest prevalence (55.5% ± 4.2). There is a significant difference between our data and the worldwide prevalence, as reported by the World Health Organization (WHO), that ranges in general population from 35% (high income countries) to 45% (low income countries) [12]. Mannino et al. [8] reported a global lower prevalence of arterial hypertension (40.1% ± 0.3) in a similar COPD population. In that study, the higher prevalence was related to higher respiratory dysfunction with a 51% rate in GOLD class 3 and 4. On the contrary, our population showed an overall higher prevalence of arterial hypertension in COPD patients with higher mean value of FEV_1_/FVC (Table 2). We observed the same result in COPD patients with diabetes. No differences were observed in lung function of COPD patients with ischaemic heart disease. A similar finding is reported by Bellocchia et al. [13] who did not find differences in pressure overload among COPD patients with higher functional impairment. Undoubtedly, a protective effect of COPD does not exist. Because our study was observational, it is more likely that patients with more severe COPD are focused on their respiratory disease instead of looking after the global health status. The same potential interpretation could be attributed to the lower value of FEV_1_/FVC in COPD patients with mood disorders, where respiratory dysfunctions drove the state of mind.

Anxiety and depression are frequent in COPD patients, because lung dysfunction prevents normal physical activities and social relationships. In the present investigation prevalence of mood disorders was 5.3% (±1.9), as reported in other studies when anxiety was present with or without depression [14, 15]. In our study, the prevalence of this disorder was significantly higher in females, because of their plausible greater frailty .

We confirm a higher prevalence of diabetes in our COPD patients (12.5% ± 2.8) than in Italian general population [16], as previously found in our pilot study [6]. It is well know that COPD patients have a higher risk of developing type II diabetes compared to normal subjects, due to the increase in circulating cytokines, in particular TNF-α, that interferes with glucose metabolism and insulin sensitivity [17].

We found the prevalence of comorbidities to be related to acute exacerbations of COPD and dyspnoea measured by MRC scale, but not with airflow obstruction. As reported by Sievi et al. [18], in COPD patients the limitation in physical activities is related to comorbidities, also in absence of FEV_1_ reduction. We evaluated the use of therapy with inhalation drugs, dividing the study population in classes of complexity, as reported in GOLD guidelines [1] for the choice of therapy: one or two bronchodilators without or in concomitance with corticosteroids.

Patients without inhalation therapy, at first respiratory evaluation, showed a moderate airflow obstruction, light but significant dyspnoea, and more than one acute exacerbation of COPD in the previous year. This phenotype represented almost 20% of the whole sample, meaning that a significant amount of sick respiratory patients remains untreated. Moreover, our study analysed respiratory function in relation to the use of inhalation drugs. We did not consider the effect of inhalation therapy on respiratory function, but only the link between therapy and respiratory conditions.As expected, a more important therapy was related to a severe respiratory impairment measured by airflow obstruction as FEV_1_/FVC and not as FEV_1_ alone. No significant correlation was evident between complexity of therapy and acute exacerbations of COPD or MRC value. Instead, a significative correlation was found between therapy and number of comorbidities, but not with Charlson Index. We suppose that in real life, when obstructive and restrictive defects frequently coexist, FEV_1_ itself is not enough to drive the therapy and it must be complemented by the FEV_1_/FVC index.

## Conclusions

According to the results of this observational study, the assessment of comorbidities to evaluate the severity of disease could be appropriate and useful in real clinical settings where it is mandatory to evaluate the global health status. However, using the number and the feature of comorbidities to drive the inhalation therapy is a still far practice in medical activity. Further studies are needed, therefore, to define the role of comorbidities and their fitting treatment in the natural history of COPD.
